# Current Status of Breast Organoid Models

**DOI:** 10.3389/fbioe.2021.745943

**Published:** 2021-11-05

**Authors:** Srivarshini Cherukupalli Mohan, Tian-Yu Lee, Armando E. Giuliano, Xiaojiang Cui

**Affiliations:** Department of Surgery, Samuel Oschin Comprehensive Cancer Institute, Cedars-Sinai Medical Center, Los Angeles, CA, United States

**Keywords:** breast organoids, 3D culture, cancer, matrix, mammary organoids

## Abstract

Breast cancer (BC) is the most frequently diagnosed malignancy among women globally. Although mouse models have been critical in advancing the knowledge of BC tumorigenesis and progression, human breast models comprising the breast tissue microenvironment are needed to help elucidate the underlying mechanisms of BC risk factors. As such, it is essential to identify an *ex vivo* human breast tissue mimetic model that can accurately pinpoint the effects of these factors in BC development. While two-dimensional models have been invaluable, they are not suitable for studying patient-specific tumor biology and drug response. Recent developments in three-dimensional (3D) models have led to the prominence of organized structures grown in a 3D environment called “organoids.” Breast organoids can accurately recapitulate the *in vivo* breast microenvironment and have been used to examine factors that affect signaling transduction, gene expression, and tissue remodeling. In this review, the applications, components, and protocols for development of breast organoids are discussed. We summarize studies that describe the utility of breast organoids, including in the study of normal mammary gland development and tumorigenesis. Finally, we provide an overview of protocols for development of breast organoids, and the advantages and disadvantages of different techniques in studies are described. The included studies have shown that breast organoids will continue to serve as a crucial platform for understanding of progression of BC tumors and the testing of novel therapeutics.

## Introduction

Globally, breast cancer (BC) is the most frequently diagnosed malignancy among women (Momenimovahed and Salehiniya 2019). As a result, much effort has been focused on understanding breast cancer tumorigenesis. However, human breast cancer development and its regulation by epigenetic and genetic changes, hormones, and external cues is still poorly understood at the molecular and cellular level. Although mouse models have been critical in advancing the knowledge of breast cancer tumorigenesis and progression, human breast models comprising the breast tissue microenvironment are needed to help elucidate the underlying mechanisms of BC risk factors. As such, it is essential to identify an *ex vivo* human breast tissue mimetic model that can accurately pinpoint the effects of these factors in breast cancer development.

Previously, two-dimensional (2D) cell cultures and cell line-derived xenograft models were commonly used to study breast cancer. While these models have been invaluable, they are not suitable for studying patient-specific tumor biology and drug response. Due to the limitation of cell-cell and cell-extracellular matrix (ECM) interactions, cell cultures grown in 2D cultures experience altered cell signaling pathways and therefore are not representative of the corresponding tissue *in vivo* (Wang et al., 1998). Cells grown in 2D monolayer conditions do not have the ability to mimic the morphology and organization of cells within tissues, and the ECM does not fully resemble that of tissues and organs (Shamir and Ewald 2014). Currently, patient-derived primary culture and xenograft models are widely used for personalized medicine research. However, patient-derived xenografts, which are produced by injecting tumor cells into the flank or mammary fat pad of mice, take months to grow and are challenging to be reproducible on a large scale (Vargo-Gogola and Rosen 2007). To address these issues, scientists have turned towards three-dimensional (3D) culture in recent decades.

The origin of 3D culture dates to the 1970s–1980s (Emerman et al., 1979; Bissell 1981). 3D culture was first attempted with normal mammary epithelial cells in collagen gels by Emerman et al., demonstrating that floating collagen gel substrates in a 3D environment provided unique factors for the growth and structural differentiation of mammary epithelial cells that plastic substrates did not provide (Emerman et al., 1979). Similarly, several studies from the 1980s echoed the notion that mammary myoepithelial cells can organize when grown in collagen gels (Flynn et al., 1982; Tonelli and Sorof 1982; Haeuptle et al., 1983). In 1992, Petersen et al. referred to these organized structures as “organoids,” or structures that result from 3D cultures (Petersen et al., 1992). Currently, the accepted definition of organoids is based on inclusion of the epithelium, given its function as the exocrine gland structure. However, the epithelium is tightly regulated by stromal components, and thus, inclusion of stromal components into organoid models would even better represent *in vivo* systems. Organoid systems are used to recapitulate several diseases, including neurodevelopmental disorders, liver conditions, colorectal cancer, prostate cancer, gastric cancer, esophageal cancer, and breast cancer (Jin et al., 2018). Breast organoids have been used to examine factors that affect signaling transduction, gene expression, and tissue remodeling (Slepicka et al., 2020). In this review, we will focus on the applications, components, and protocols for development of breast organoids.

### Applications of Breast Organoids

Studies have demonstrated the multipurpose nature of mammary organoids, especially in the analysis of mammary gland development (Jamieson, Dekkers et al., 2017). Organotypic culture has been utilized to observe mammary ductal elongation over time and identify molecular pathways that contributed to collective cell movement (Ewald, Brenot et al., 2008; Huebner et al., 2016). Simian et al. suggested that matrix metalloproteinases are an essential factor in mammary branching morphogenesis through the use of mouse mammary epithelial organoids, in which organoids were prepared from both 10-week-old CD-1 mouse tissue and a normal mouse mammary epithelial cell line (Simian, Hirai et al., 2001). With epithelial organoids isolated from mammary glands of pubertal mice, Sumbal and Koledova showed that fibroblasts regulate branching of mammary epithelium (Sumbal and Koledova 2019). Zhang et al. similarly showed that different FGF ligands are involved in regulation of epithelial behavior utilizing mouse mammary organoids (Zhang, Martinez et al., 2014). Xian et al. concurred with the finding that 3D culture can be employed in understanding tissue response to growth factors in their study of FGF receptors in an immortalized murine mammary epithelial cell line (Xian et al., 2005). Furthermore, 3D culture is useful in the examination of types of progenitor cells in the breast, as demonstrated by studies utilizing normal human mammary epithelial organoids (Villadsen et al., 2007; Fridriksdottir et al., 2017). Moreover, hormones can be included to create models of physiological processes. Sumbal et al. produced a model of lactation and involution by exposing epithelial organoids derived from fresh mouse tissue to pregnancy hormones (Sumbal et al., 2020). Davaadelger et al. analyzed breast organoids grown from BRCA1 mutant human mammary tissue that were then exposed to estradiol and progesterone, showing specifically that the progesterone receptor activity is different from non-carrier organoids (Davaadelger et al., 2019). Organoids have also helped elucidate the role of epithelial-stromal interactions during mammary branching morphogenesis (Nguyen-Ngoc and Ewald 2013).

Accordingly, organoids have been useful in the current understanding of normal mammary gland development, and can also been utilized in the study of abnormal cell processes.

Importantly, breast organoids can be used as models for disease (Jin et al., 2018). They can be easily manipulated, enabling detailed study of cell-cell and cell-ECM interactions (Huch and Koo 2015). Additionally, recent studies have shown that breast tumor organoids are able to express the heterogeneity of cancer subtypes when grown in 3D culture, which is particularly useful when studying the effect of therapeutics. Sachs et al. developed >100 primary and metastatic BC organoid lines from human breast tumor tissue; notably, the majority of these BC organoids matched their original BC tumor in histopathology, hormone receptor status, and HER2 receptor status (Sachs et al., 2018). Sachs et al. also suggested that future studies should collect tumor RNA to distinguish the influence of tumor environment on gene expression of BC cells (Sachs et al., 2018). Breast organoids are valuable in the study of carcinogenesis as well. Lee et al. used epithelial organoids derived from biopsies of human primary breast carcinomas to show that their expression of an Na (+)HCO3(-) cotransporter, which has been associated with increased BC susceptibility, was higher than that of matched normal organoids from the same patients (Lee et al., 2015). Bischel et al. developed a scalable 3D model of ductal carcinoma *in situ* (DCIS) that can be incorporated to study drug treatment by filling mammary epithelial cell-lined lumens with DCIS cells to create a DCIS-like structure (Bischel et al., 2015). Recently, Dekkers et al. modeled BC using breast organoids that were derived from normal breast tissue and genetically engineered with CRISPR/Cas9, recapitulating oncogenesis. Four BC-associated tumor suppressor genes were knocked-out with CRISPR/Cas9, and organoids that had long-term culturing ability and responded to therapeutics were developed (Dekkers et al., 2020).

### Matrix of Breast Organoids

Organoids are incorporated into matrices that contain basement membrane proteins essential for epithelial cell function and polarization. Placement into these matrices enables cells to organize into 3D structures *in vitro* (Shamir and Ewald 2014; Gilpin and Yang 2017; Nayak et al., 2019; Slepicka et al., 2020). Matrices contain points of attachment for the cells and enable proper transport of nutrients and other essential ingredients to the cell. The major ECM components include matrix proteins, glycosaminoglycans, proteoglycans, glycoproteins, growth factors, and other secreted proteins (Hynes and Naba 2012). Critically, the matrix must resemble the stiffness of the natural tissue (Gilpin and Yang 2017). Matrix stiffness is an important feature as it alters epithelial morphogenesis by clustering integrins and regulates cell fate by modulating growth factor signaling and Rho GTPase function (Paszek et al., 2005).

Current matrices include both natural and synthetic scaffolds. Matrigel and collagen I are two common natural scaffolds. Commercially available Matrigel, which is Engelbreth-Holm-Swarm ECM extract, is the most frequently used matrix gel, and it is rich in ECM proteins like laminin, collagen IV, entactin/nidogen, heparan sulfate proteoglycans, and growth factors.

Interestingly, Nguyen-Ngoc et al. utilized different ECM gels to test the importance of the ECM in tumor cell dissemination and discovered that in Matrigel, malignant epithelium grew without protrusions whereas in collagen I, epithelium from the same tumor promoted an invasive phenotype (Nguyen-Ngoc et al., 2012). As such, cell signaling and gene expression patterns may be influenced by and dependent on the particular matrix chosen, illuminating the importance of the ECM components. Utilizing the best ratio of Matrigel and collagen I may be necessary in order to accurately model human mammary branching morphogenesis. In a later study, Nguyen-Ngoc et al. found that organoids in mixed gels of Matrigel and collagen I, as opposed to just Matrigel or just collagen I, provided a more accurate model of mammary branching morphogenesis *in vivo* (Nguyen-Ngoc and Ewald 2013). The concentration of the collagen I fibrils in the mixed gel was of high importance as well. Therefore, the proper composition of ECM should be carefully determined in order to create a human breast model comprising the breast tissue microenvironment.

Much less commonly utilized in breast organoid culture, synthetic scaffolds include polymers that retain the mechanical properties of the tumors, such as polyethylene glycol, polyvinyl alcohol, polycaprolactone, and polylactide-co-glycolide, although their stiffness may not be as similar to *in vivo* tissue as that of naturally derived scaffolds (Rimann and Graf-Hausner 2012).

### Discussion of Breast Organoid Protocols

**FIGURE 1:**
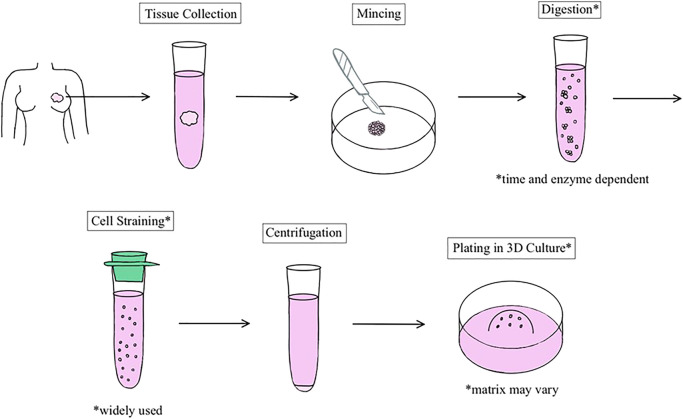
Schematic representation of the general process of human breast organoid derivation. Organoids are established from resections of normal breast or tumor tissue.

Many breast organoid protocols have been developed with the main variations consisting of differences in matrix type, medium components, and plating techniques (Table 1). The first assay to derive organoids from fresh tissue arose from studies published in the Bissell and Werb groups (Simian, Hirai et al., 2001; Ewald, Brenot et al., 2008). Currently, as described by Mazzucchelli et al. for patient-derived BC organoids, this methodology involves 1) tissue digestion, 2) suspension in Matrigel and application of growth medium, 3) passage of organoids once they become too large or too numerous, and 4) paraffin embedding for immunohistochemistry and histology. The advantage of this protocol was its ability to develop enough replicable organoids to develop a biobank (Mazzucchelli et al., 2019). Chen et al. also added to this protocol to describe the steps required to isolate normal mammary epithelial stem cells from fresh human breast tissue. This methodology also describes the steps entailing the stem cells’ differentiation and passage in 3D organoid culture (Chen et al., 2019). In general, most protocols follow a similar sequence of steps (Figure 1), albeit technical or component modifications may be included. Examples of these variations include plating organoids in only collagen I versus a mixture of Matrigel and collagen I. Nguyen et al. described a protocol for isolating epithelial organoids from normal mouse mammary glands, and pointed out that mixed Matrigel and collagen I matrices may represent a more physiological ECM microenvironment (Nguyen-Ngoc et al., 2015). Growth factors such as epidermal growth factor or fibroblast growth factor 7, molecular inhibitors such as Y-27632 that help tumor cells proliferate *in vitro*, and other regulators of signaling pathways are added to generate growth and differentiation of organoids (Yu and Huang 2020). Unique components such as the mitogen Neuregulin 1 may be added to efficiently generate BC organoids and long-term expansion for subsequent passages, as illustrated by Sachs et al. (2018) It is important to note that while these organoids retain their distinct molecular subtypes, general long-term culture may result in molecular and phenotypic drift (Goldhammer et al., 2019).

**TABLE 1:** Significant mammary organoid studies with associated protocol details.

Study (Year)	Source of organoid	Type of matrix	Technique	Significance of study
Simian et al. (2001)	Normal mouse mammary glands and mammary epithelial cell lines	Type I Collagen	Organoids embedded in collagen gels	Provided evidence for role of matrix metalloproteinases in mammary epithelial branching
Lee et al. (2007)	Human breast cell lines, either normal or malignant	Engelbreth-Holm-Swarm extracellular matrix extract, Matrigel	Embedded assay with layering method, 3D on-top assay	Describes different techniques for 3D culture assays
Ewald et al. (2008)	Transgenic mouse mammary glands	Matrigel	Suspension of organoids in Matrigel	Demonstrated common branching mechanisms of branching morphogenesis in mammary epithelial cells
Nguyen-Ngoc et al. (2015)	Normal mouse mammary glands	Both Matrigel and Collagen I	Several protocols for different ECMs	Provides examples of outcomes of various types of assays with different ECMs
Sachs et al. (2018)	Human breast cancer tissue	Basement Membrane Extract	Cells suspended in basement membrane extract drops	Established a biobank of breast cancer organoids
Djomehri et al. (2019)	Malignant mouse mammary tissue, immortalized cell lines	Matrigel	Hanging drop array	Demonstrated scaffold-free technique
Mazzucchelli et al. (2019)	Human breast cancer tissue	Matrigel	Seeding of organoids in Matrigel droplets	Described novel technique for isolating patient-derived organoids from surgical and biopsy specimens
Chen et al. (2019)	Normal human breast tissue	Matrigel	Growth of organoids from mammospheres in low-attachment plates	Described isolation of progenitor cell-generated organoids
Mollica et al. (2019)	Normal human and rat mammary tissue	Decellularized rat or human breast tissue utilized to form hydrogels	Bioprinting technique	Developed 3D bioprinted organoids with significant reproducibility
Dekkers et al. (2021)	Human normal and breast cancer tissue	Basement Membrane Extract	Tissue suspended in basement membrane extract and plated in drops	Demonstrated long-term culture of normal and breast cancer organoids
Pan et al. (2021)	Human malignant pleural effusion	Basement Membrane Extract	Pleural effusion specimen centrifuged, suspended in basement membrane extract, and placed suspension plate	Successfully expanded pleural effusion-derived triple negative BC organoids

In addition to utilizing different types of matrices and media components, there are several layering methods that can be used in the development of breast organoids. The single layer technique entails cells being mixed directly with the matrix and polymerized in one thick layer. In their protocol, Lee et al. describe a 3D embedded assay in which they coat a culture surface with a thin layer of ECM extract before plating breast cancer cells resuspended in ECM extract (Lee et al., 2007). The advantage of adding a thin layer of ECM extract prior to plating is that the layer prevents the cells from sinking and attaching to the bottom of the culture surface. As seen by Campaner et al., without the layer of matrix at the bottom of the wells, the BC organoids gradually invaded the matrix and sank into the plate, resulting in the loss of the organoids’ 3D structure and eventually the organoids themselves (Campaner, Zannini et al., 2020). Multi-layered techniques can induce the formation of different phenotypes, resembling *in vivo* environments in which the inner portion of tumors are not as vascularized and receive less nutrients. Multi-layering methods also enable co-culturing of various cell types (Ibarrola-Villava et al., 2018). Aside from the 3D embedded assay, Lee et al. also developed the 3D on-top assay, in which cells are cultured on top of a thin ECM gel that is overlaid with a dilute solution of ECM (Lee et al., 2007). This technique is less labor-intensive, more cost-effective, and facilitates imaging in a single plane. However, it is not as ideal for studying cell-cell interactions. Finally, the dome formation method suspends cells in droplets that contain ECM extract onto a surface, which enables media exchanges and addition of chemicals more easily (Djomehri et al., 2019). For example, Sachs et al. isolated BC cells from BC tissue and resuspended them in basement membrane extract drops that were then plated and overlaid in their BC organoid culture medium. Using the same protocol, Rosenbluth et al. were able to demonstrate that this long-term culture method preserved the complex stem/progenitor and differentiated cell types of the breast (Rosenbluth et al., 2020). Dekkers et al. also followed a similar technique in which they resuspended digested normal breast and BC tissue in basement membrane extract, plated the basement membrane extract-containing cells in multiple small drops, and then supplemented with expansion medium (Dekkers Vliet et al., 2021). With their optimized protocol, Dekkers et al. generated a biobank of BC organoids that typically recapitulated the original patient tumor, and demonstrated the ability for long term culture and genetic tractability for both normal and BC organoids.

The ECM scaffold provides a structure to guide cell organization. Scaffolds such as Matrigel remain the most prominent matrix, but some scientists utilize decellularization strategies to remove native cells and genetic material from a naturally derived ECM and personalize it with the patient’s own cells. Gilpin and Yang described the advantages and disadvantages of chemical and enzymatic, physical, or combination methods for decellularization (Gilpin and Yang 2017). Chemical agents include surfactants, acids, bases, and enzymes, which either cause cell lysis or solubilize the cell membrane. These agents can efficiently remove cells and genetic material, but some require extensive wash processes due to their toxicity and can cause clumping of DNA (Gilpin and Yang 2017). Mechanical decellularization methods include high hydrostatic pressure, supercritical carbon dioxide, and freeze-thaw; these methods mitigate the concern of toxicity, but also can leave remnant host DNA fragments. Protocols utilizing decellularization to grow organoids in other organ systems have been developed but are infrequent in the realm of breast organoids specifically. Although ECM scaffolds help provide structure, they may also trap growth factors, cytokines, and chemokines, masking the full impact of therapeutics. Djomehri et al. used a liquid-based system to develop a scaffold-free organoid model. In their study, organoids were developed from BC mouse tissue; these organoids maintained the spindle cell morphology of the primary tumors. Djomehri et al. suggested this could be a better platform for testing therapeutics and studying tumorigenic signaling, although the model is difficult to maintain long-term, more time consuming, and has a risk of droplet dehydration (Djomehri et al., 2019).

Recent studies have also suggested methods to improve the reproducibility of organoid development by utilizing 3D bioprinting. Reid et al. explained the benefits of 3D bioprinting in a study that utilized immortalized non-tumorigenic human breast epithelial cell lines and a collagen I matrix. Results included the ability to form organoids from very few cells, increased efficiency, and greater control over the location of cells within the gel (Reid et al., 2018; Reid et al., 2019). Mollica et al. also utilized a 3D bioprinter to create a hydrogel consisting only of ECM from decellularized rat or human tissue (Mollica et al., 2019). This hydrogel was compared to standard Matrigel and collagen matrices; BC cell lines demonstrated unique properties and growth responses when grown in the hydrogel. One limitation of this technique was the inability of the deconstructed matrix to form structures with the same order necessary for complete biomimicry, as organoids may not recapitulate specific fiber orientations exactly.

### Limitations of Current Breast Organoid Models

It is evident that breast organoid technology is revolutionizing the study of normal human breast development as well as tumorigenesis. However, there are still limited breast organoid models that have been able to recapitulate native breast tissue ECM composition or fiber structure. It is important to note that many current protocols involve several steps of differential centrifugation and cell straining in order to separate and purify mammary epithelial cells from surrounding stromal cells before growth in 3D culture. Using purified epithelial cells and stromal cells to produce breast organoids may not recapitulate the *in vivo* tissue architecture and cell-cell/matrix interactions.

Further research should be performed to incorporate multiple cell types in breast organoid models. Although epithelium is only required in the current accepted definition of organoids, incorporation of other cell types may contribute to a more accurate recapitulation of *in vivo* breast architecture. Rosenbluth et al. successfully included stromal cells in their breast organoid model but showed that after several passages, stromal cells were lost. The differences in epithelial structure and cell populations between the resulting organoids and matched human breast tissue was in part attributed to the absence of stromal cells in passaged organoids, which demonstrates the significance of stromal cells in these models (Rosenbluth et al., 2020). Davaadelger et al. also included fibroblasts in organoids to study the effects of BRCA1 mutations on progesterone response in breast cells (Davaadelger et al., 2019). Other studies have successfully co-cultured organoids with stromal cells. Hacker et al. developed organoids from normal and irradiated mouse mammary gland tissue that interacted with macrophages in co-culture experiments (Hacker et al., 2019). Moreover, Truong et al. created a 3D organotypic microfluidic tumor model and co-cultured BC and patient-derived fibroblast cells to examine the role of cancer-associated fibroblasts in tumorigenesis (Truong et al., 2019). Continued research with these types of studies may help bring organoid models one step closer to mimicking the native breast microenvironment.

## Conclusion

This review paper summarizes the multiple applications of breast organoids and provides an overview of the protocols that are used to generate these organoids. Breast organoids have significant utility in understanding not only normal human mammary gland development, but also breast tumor development. Future directions will include addressing updates on breast organoid technology as well as continuing to incorporate multiple cell types into organoid models. Breast organoids will continue to serve as a crucial platform for understanding of the progression of BC tumors and the testing of novel therapeutics.

## Abbreviations

BC, breast cancer; 2D, two-dimensional; ECM, extracellular matrix; 3D, three-dimensional; iPSCs, induced pluripotent stem cells; FGF, fibroblast growth factors; DCIS, ductal carcinoma *in situ*.
